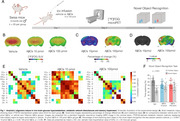# Impact of beta‐amyloid oligomers on in vivo brain glucose metabolism

**DOI:** 10.1002/alz.095067

**Published:** 2025-01-09

**Authors:** Andreia Silva da Rocha, Igor C. Fontana, Guilherme G. Schu Peixoto, Amanda S Souza, Gianina Teribele Venturin, Samuel Greggio, Leticia Forny‐Germano, Mychael V Lourenco, Pedro Rosa‐Neto, Diogo O. Souza, Jaderson Costa da Costa, Sergio T Ferreira, Eduardo R. Zimmer

**Affiliations:** ^1^ Universidade Federal do Rio Grande do Sul, Porto Alegre Brazil; ^2^ University of Pittsburgh, Pittsburgh, PA USA; ^3^ Federal University of Rio de Janeiro, Rio de Janeiro Brazil; ^4^ Brain Institute of Rio Grande do Sul, Porto Alegre Brazil; ^5^ McGill University, Montreal, QC Canada; ^6^ Federal University of Rio de Janeiro, Rio de Janeiro, RJ Brazil; ^7^ D'Or Institute for Research and Education, Rio de Janeiro, RJ Brazil

## Abstract

**Background:**

The deposition of β‐amyloid (Aβ) plaques is a classical neuropathological feature of Alzheimer’s disease (AD). Currently, it is believed that intermediate products of the Aβ fibrillogenesis process, like the β‐amyloid oligomers (AβOs), are the most toxic forms, and are involved in neurodegenerative processes in AD. The evaluation of cerebral glucose metabolism in patients with β‐amyloid plaque deposition using [^18^F]FDG‐PET has been used as a marker of neurodegeneration in AD. However, little is understood about AβOs' impact on glucose metabolism prior to Aβ plaques formation. The aim of this study was to evaluate the impact of the intracerebroventricular infusion of AβOs on in vivo glucose metabolism via [^18^F]FDG‐PET.

**Method:**

Male Swiss mice (3‐month‐old, n = 20 per group) were divided into three groups: Vehicle, AβOs 10pmol, and AβOs 100pmol. Vehicle/AβOs were infused into mice’ right ventricle using the freehand technique after brief isoflurane anesthesia. [^18^F]FDG‐PET scans were performed 24h after AβOs infusion. The same animals underwent the Novel Object Recognition (NOR) task 24h after scanning. The images were processed and analyzed using MINC tools. Metabolic networks were built by computing Pearson correlation coefficients based on 2,000 bootstrap samples and FDR corrected (P<0.05). See **Fig. 1a,** for a experimental design illustration.

**Results:**

AβOs induced dose‐dependent brain glucose hypometabolism (**Fig. 1b**) and metabolic network disturbances. Voxel‐wise percentage change analysis revealed moderate reductions in glucose brain metabolism (5% to 15%) following infusion of 10pmol AβOs, whereas 100 pmol AβOs led to widespread and substantial reductions of up to 25% (**Fig. 1c**). T‐statistical voxel‐wise analysis indicated statistically significant brain glucose hypometabolism only in the 100pmol group (**Fig. 1d**). The low‐dose AβOs (10pmol) induced brain inter‐region metabolic hyperconnectivity, while the 100 pmol dose caused hypoconnectivity (**Fig. 1e**). Furthermore, both AβOs infusion groups exhibited impaired recognition memory in the NOR task (**Fig. 1f**).

**Conclusion:**

Our results are the first demonstration of AβOs causing in vivo glucose hypometabolism and metabolic network disturbances in the absence of plaques. These findings point to an early impact of AβOs on glucose metabolism, independent of β‐amyloid plaque formation. While the molecular pathways underlying this effect require further investigation, they may represent important AD pathophysiological mechanisms and potential targets for therapeutic intervention.